# Smallpox and Vaccination in Siam

**Published:** 1915-02-06

**Authors:** 


### SMALLPOX AND VACCINATION IN SIAM.

### A Remarkable Educational Campaign.

A singularly interesting report has recently been issued by Dr. Campbell Highet, principal medical officer to the Local Government Board in Siam. The point of departure of the report is the promulgation of a law making the practice of vaccination compulsory in the "monthon" of Bangkok, and rendering possible the establishment by proclamation of the same claim in other parts of the kingdom. Dr. Highet has taken the opportunity thus afforded to present a brief history of smallpox and of vaccination in the country which he so well serves as guardian of the public health. Like other Eastern countries Siam has for long suffered from frequent epidemics of smallpox, and no systematic scientific efforts to cope with the disease can be traced prior to the year 1838. At that time an epidemic of unusual severity was raging, and some American missionaries, with a view to protect their own children from the scourge, inoculated them with the virus of the disease. Fortunately, in every instance the result was a favourable one, and the story coming to the ear of the King, his Majesty ordered a full investigation by the royal physicians. As a result inoculation became widely practised, more especially perhaps among the upper classes, and as an evidence of its success it is mentioned that probably not more than one case in 500 proved fatal. Indeed, so widespread was the confidence felt in inoculation that there was a general reluctance to abandon it for any other method.

### The Infectious Diseases Hospital.

In 1840 some active vaccine matter was brought to Siam from the United States, and a number of children were vaccinated. The practice, however, could not be steadily maintained, as the virus gradually lost its efficacy and fresh supplies were obtained only with difficulty and at irregular intervals. But in 1904 the Siamese Government itself commenced the preparation of calf lymph at Bangkok, and this is continued at the official Pasteur Institute under the direction of Dr. Manaud and Dr. Roberts; during the last seven years nearly two million doses of vaccine lymph have been issued to the public. Other efforts to popularise the practice have included the training and certification of sanitary inspectors and other suitable officials; the institution of an educational campaign in all parts of the country at the wish and expense of his Majesty the King; and the establishment of special stations in hospitals, temples, and police stations, where free vaccination can be obtained. In spite of these various measures and of the improvement which followed their adoption, smallpox still continued to number many victims. The deaths during the epidemic of 1911-12 amounted to 3,308, that is a mortality of 4.3 per thousand of the population. As an indication of the virulence of the disease Dr. Highet quotes some figures from the Hospital for Infectious Diseases, and these show a death-rate of 55.9 per cent. of those attacked. From another table we learn that 25 per cent. of the deaths occurred in infants under one year and 67.2 per cent. in children under ten years of age. Thus a review of the whole position forced the conclusion that while voluntary vaccination had produced great benefits, it was not sufficient to meet fully the demands of the situation—that, in short, it left, even in Bangkok, large numbers of unprotected persons. And as this was true of Bangkok, it was true in still greater measure of the interior of the country.

### Comparison of East and West.

From what is here related it is apparent that the new Act enforcing compulsory vaccination has not been adopted in any hasty or arbitrary spirit. It rests rather on a solid body of practical experience, which has convinced the authorities, first, of the value of vaccination, and, secondly, of the necessity for compulsion if the full measure of individual and national advantage is to be secured. The provisions of the measure closely follow legislative enactments with which we are familiar nearer home. Provision is made for free vaccination at suitable stations and for the licensing of competent officials; no lymph may be used other than that authorised by the Government; and the practice of inoculating with the virus of smallpox is forbidden under heavy penalties. In two respects the Act departs from its British model. First, in one of the clauses authority is given for the issue of a public notice enforcing not merely vaccination, but also revaccination, upon the inhabitants of any specified district or locality in which there is, or is likely to be, an epidemic of smallpox. Secondly, the Act knows not the "conscientious objector," who thus remains a pure product of our Western civilisation. Whether on this Siam deserves congratulation or sympathy is a matter on which opinions may possibly differ. Finally, we will venture in a word to congratulate Dr. Campbell Highet both on his report and on the work of which it is one of the products. We have long been familiar with the effective fashion in which he has helped to carry the beneficial influences of preventive medicine into the homes of the people of Siam, and we cordially wish him a continued and an increasing success.

In the above connection it is interesting to note that the King of Siam has conferred on Dr. Campbell Highet the Order of the White Elephant. The *cachet* attaching to this decoration, somewhat hard to realise in London, needs no insisting on by all who have travelled east of Suez.

